# Genetically Engineered Yeast for Enhanced Biodegradation of Β-lactam Antibiotics

**DOI:** 10.1007/s12010-025-05291-4

**Published:** 2025-06-27

**Authors:** Carolin Pohl, Cindy Rau, Linda Schuster, Uta Gutbier, Stephan Beil, Katrin Lehmann, Hilmar Börnick, Kai Ostermann, Stefan Stolte

**Affiliations:** 1 Faculty of Environmental Science, Institute of Water Chemistry, TUD Dresden University of Technology, Dresden, 01062 Germany; 2 Faculty of Civil Engineering, Division of Water Sciences, HTW University of Applied Sciences, Friedrich-List-Platz 1, Dresden, 01069 Germany; 3 Else Kröner Fresenius Center for Digital Health, Faculty of Medicine Carl Gustav Carus, TUD Dresden University of Technology, Dresden, Germany; 4 Faculty of Biology, Research Group Biological Sensor-Actuator-Systems, TUD Dresden University of Technology, Dresden, 01062 Germany

**Keywords:** Antibiotics, Enzymatic degradation, LC–MS/MS, β-lactamase

## Abstract

High environmental concentrations of pharmaceuticals, particularly antibiotics, have been observed worldwide, while antibiotic concentrations in the ng/L to µg/L range may adversely affect biota and contribute to the formation of antibiotic resistance. The underlying causes of high environmental concentrations include, in addition to the low rate of antibiotic’ metabolization and the high rate of usage, especially of β-lactam antibiotics, the insufficient removal by conventional wastewater treatment methods. Consequently, alternative methods need to be developed to remove antibiotics from wastewater—one possibility is the use of enzymes. In this study, the enzyme β-lactamase was secreted by a genetically modified yeast (*Saccharomyces cerevisiae*) upon recognition of a pheromone (α-factor) as inducer to enable the degradation of ampicillin. This represents a crucial step on the road to a sensor-actuator system, allowing for the development of an intelligent removal system that can react to the presence of antibiotics. Ampicillin and its transformation products were studied by LC–MS/MS measurements using a carbamoyl functionalized column under hydrophilic interaction liquid chromatography (HILIC) conditions, which allowed detection of ampicillin at concentrations of 2.43 nM. The dependence of ampicillin degradation on α-factor concentration and the cultivation time of the yeast was demonstrated, resulting in higher degradation rate with higher α-factor concentrations and longer yeast cultivation times. Over 90% of 10 µM ampicillin was degraded within 0.5 h using 250 nM α-factor and a cultivation period of 24 h. Finally, the transferability to other β-lactam antibiotics was investigated, resulting in complete degradation of amoxicillin, penicillin G and piperacillin within 24 h.

**Supplementary Information:**

The online version contains supplementary material available at 10.1007/s12010-025-05291-4.

## Introduction

Antibiotics account for approximately 70% of pharmaceuticals used in human and veterinary medicine [1]. Annually, the global production of antibiotics is estimated to range between 100,000–200,000 tons [2], with a steady increase in forthcoming years in response to the global demand, which is estimated to rise by approximately 50% by 2030 [3]. There are more than 20 different antibiotic classes [4] with the most frequently used being β-lactams followed by cephalosporine, macrolides, and fluoroquinolone [5]. These antibiotics are continuously being released into the environment because they cannot be completely metabolised in animals (reviewed in [6]) or in the human body (reviewed in [7]), are not completely removed in common wastewater treatment plants (WWTP) (reviewed in [8, 9]), and, in the case of hydrophilic compounds, can have a high mobility within the environment [10]. Besides this, the high usage and the persistence or pseudo-persistence of many antibiotics lead to increasing environmental concentrations (review [11, 12]). There, antibiotics may affect organisms directly or indirectly [13] and can adversely affect flora and fauna worldwide [14], regardless of their point of entry (reviewed in [15]). Antibiotics influenced the microbial community composition, have negative impact on reproduction and growth of fish [8] and also phytotoxic properties concerning plant growth and development [13]. Of major concern is the possibility that antibiotics in the environment may contribute to the development of antibiotic resistance even at long-term exposure to concentrations of antibiotics in the ng/L range as reported elsewhere [8, 16–18]. The world health organisation (WHO) has identified bacterial resistance as one of the most significant challenges and threats to both global health and the economy [19]. It is estimated that there were 5 million deaths related to antibiotic resistance in 2019 [20]. Consequently, it is imperative that policies and regulations are implemented to control and mitigate the anthropogenic discharge of antibiotics into the environment [15].

Antibiotics are typically classified based on their structural characteristics and mode of action [21], with β-lactam antibiotics, in addition to classes of macrolides, tetracyclines, quinolones, and sulphonamides, being among the most frequently used [22]. In some cases, the half-life of β-lactams is longer than the retention time in wastewater treatment plants [23] and only 73–77% of β-lactam antibiotics were found to be removed during wastewater treatment (reviewed in [9]). For example, the half-life of amoxicillin is reported to range from three hours to more than five days ([24]). Therefore, high concentrations ranging from 10 ng/L to a maximum of 1 µg/L (reviewed in [9]) of β-lactams such as ampicillin, amoxicillin and cloxacillin can be found in wastewater treatment plant effluents in Europe, Asia, Africa and North America. Therefore, one promising strategy to reduce the environmental concentrations of β-lactams is to improve WWTP efficiency through the addition of further purification stages, as reducing the consumption of antibiotics by itself, will not suffice (reviewed in [25]).

For instance for the commonly prescribed β-lactam ampicillin (AMP), granular activated carbon can remove 73% of 20 mg/L AMP within 2 h [16], while Elmolla and Chaudhuri’s UV/ZnO photocatalytic process takes approximately 3 h to remove 97.3% of 105 mg/L AMP at pH 11 [26]. Furthermore, a photo electro-Fenton process can remove 90% of 50 mg/L AMP within 2 h [27]. Watkinson et al. have achieved an overall removal rate of 94% using a combination of microfiltration and reverse osmosis [28]. However, these physicochemical processes are very costly due to the energy and chemicals required (reviewed in [8]). A promising new technology with the potential to remove sufficient quantities of antibiotics while offering a low-cost and easy-to-implement solution that is free of toxic-by-products (reviewed in [8] and [29]) is the use of enzymes. Enzymes has already been successfully tested to remove other pharmaceuticals such as Diclofenac, 17-β-estradiol and Triclosan (see review [29] and [30–32]), subsequently a concept with broad applicability.

**Fig. 1:**
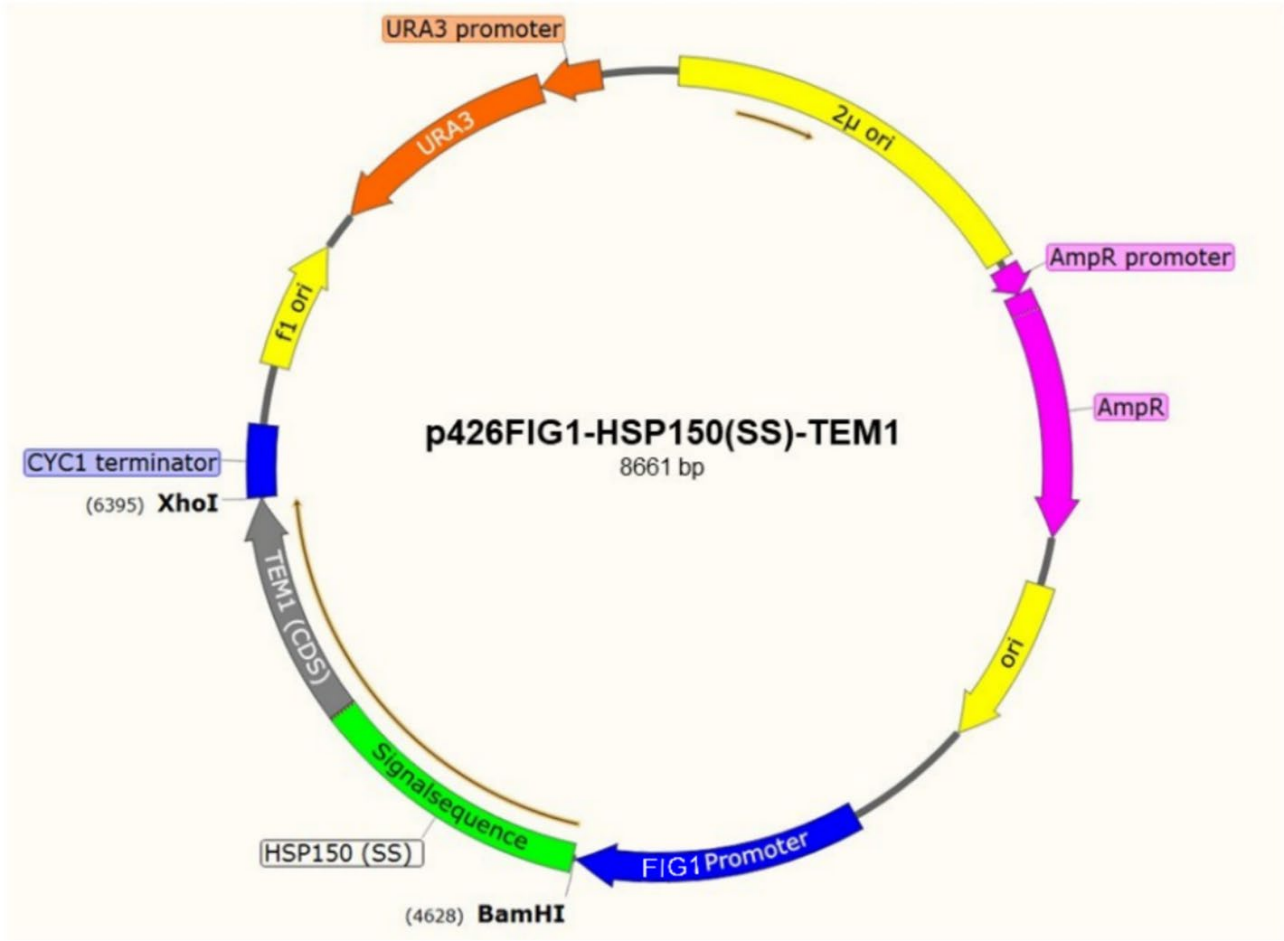
Scheme of the yeast plasmid p426-FIG1-HSP150(SS)-TEM1. The β-lactamase coding sequence (*TEM1*) was fused to a secretion signal (*HSP150(SS)*) and set under the control of the α-factor dependent *FIG1* promotor. The construct was integrated into the high-copy vector p426 [37] to facilitate a high-level production and secretion of β-lactamase upon addition of α-factor

This study will present a novel system for the inducible production and secretion of the enzyme β-lactamase (TEM1p). It is part of an overall vision that aims to establish cross-kingdom cell–cell communication between bacterial and yeast cells as a sensor-actuator system that can be used in the treatment of process water. Ultimately, in our vision, genetically modified bacteria will act as sensors (here of an antimicrobial compound) and in response will produce a yeast pheromone (e.g. α-factor) that triggers the expression of, in this example, lactamases in the yeast actuator cells, resulting in AMP degradation. For this purpose, the baker's yeast *Saccharomyces* (*S.) cerevisiae* is employed, which is widely used for the low-cost and rapid production of substances due to its simple metabolism, ease of genetic modification and robustness (see review [33] and [34]). The integration of the *TEM1* gene under the control of the α-factor sensitive FIG1 promoter in the high-copy expression vector p426FIG1 enables the yeast to respond by producing and secreting β-lactamase in the presence of the yeast-specific α-factor. The β-lactamase produced, hydrolyses the β-lactam ring, thereby resulting in a loss of antibiotic activity as already mentioned by [35, 36].

Thus, it is necessary to develop a sensitive (minimum LOD of 10 nM) and robust LC–MS/MS method to monitor AMP degradation in culture media without further sample preparation. Western blot analysis will initially be used to confirm the enzyme production and secretion, while the AMP degradation from supernatants will be directly analysed by LC–MS and compared with the results of the non-specific lactam quantification via the nitrocefin assay. Subsequently, the methodology will be tested for its applicability to the β-lactam antibiotics: amoxicillin (AMX); cloxacillin (CLX); penicillin G (PEN G); cefalotin (CET) and piperacillin (PIP).

## Materials and Methods

### Generation of Genetically Modified Yeast and Cultivation

For the generation of a *TEM1* expression plasmid, the *S. cerevisiae* vector p426 (high copy number 2*µ* vector) has been used [37]. The AmpR gene in the plasmid backbone (see Fig. 1) is exploited for cloning procedures in bacterial strains and will not be active in yeast. The promoter FIG1 was cloned into the vector to generate the plasmid p426FIG1 [38]. The target gene *TEM1*, fused to the secretion signal sequence from HSP150 (*HSP150(SS)* [39]*;*) (Fig. S1), was synthesized by BioCat GmbH (Germany). The construct was integrated downstream of the FIG1 promoter to facilitate α-factor induced production and secretion of β-lactamase.

For expression and secretion of TEM1p, the *S. cerevisiae* strain BY4741Δ*bar1* (Euroscarf Acc. no Y01408) was used. The deletion of the *BAR1* gene, which encodes a protease that can degrade α-factor [40], ensures α-factor stability and a long-term induction of heterologous gene expression. The expression plasmid p426-FIG1-HSP150(SS)-*TEM1* (Fig. 1) was transformed into BY4741Δ*bar1* using the lithium acetate/single-stranded carrier DNA/polyethylene glycol method [41].

The yeast strain was cultivated in W0 medium (1.7 g/L yeast nitrogen base, 5.0 g/L ammonium sulfate, 20 g/L glucose) supplemented with L-histidine (60 mg/L), L-leucine (80 mg/L) and L-methionine (20 mg/L). The media components were purchased from Formedium Ltd. (United Kingdom). Cultivation was performed at 30 °C with constant agitation at 180 rpm.

For the production and secretion of TEM1p, shaken flask cultures were inoculated with overnight cultures of the expression strain to an OD_600_ of 1.0. Immediately, expression was initiated by the addition of synthetic α-factor (MOLSURF GmbH & Co. KG, Germany; amino acid sequence WHWLQLKPQPMY) to final concentrations of 10, 50, 100 and 250 nM. A control culture without α-factor was run in parallel for each experiment. Samples were taken 0, 2, 4, 6 and 24 h after induction. Yeast cells were centrifuged at 3,500 × g for 5 min and supernatants were used for the various enzyme activity analyses. If necessary, samples were stored at −20 °C until further analysis.

### Western Blot Analysis

For expression analysis, samples were taken 0, 2, 4, 6 and 24 h after induction with α-factor and centrifuged at 5000 × g for 5 min. Protein extracts from cells were prepared as described previously [42]. The supernatants were concentrated to 1/10 initial volume using Vivaspin Turbo 4 10 K (Sartorius AG, Germany).

Proteins (20 µg per lane) were separated by SDS-PAGE according to Laemmli [43] using 4% stacking gels and 12% running gels. Proteins were transferred by semi-dry Western Blot to an Immobilon-P polyvinylidene fluoride membrane (0.45 µm, Merck Millipore) after the gel was run and probed with HRP-coupled antibodies directed against TEM1p (Flarebio Biotech LLC., USA). Detection was performed using the Western-Bright Kit (Biozym Scientific GmbH, Germany). Coomassie staining of the proteins in the polyacrylamide gel was performed according to Neuhoff et al. [44].

### Nitrocefin-Assay

Nitrocefin is a chromogenic cephalosporin whose absorption maximum shifts from 390 to 486 nm when the β-lactam ring is hydrolytically cleaved. For each sample, 500 µL of each culture to be analysed were centrifuged at 5000 × g and 4 °C for 10 min. The cell-free supernatant was used for enzyme activity assays. Briefly, 80 µL of the 1:5 in water diluted supernatant was mixed with 20 µL Nitrocefin working solution (5% (v/v) Nitrocefin (in DMSO), 95% (v/v) potassium phosphate buffer (0.1 M, pH 7.4)). The assay was set up in a 96-well plate (Greiner, Germany) and incubated for 30 min at room temperature in the dark before measuring absorbance at λ = 486 nm (TECAN multifunction reader, Switzerland). Data represent the mean value and standard deviation of triplicates of three independent experiments.

### LC–MS/MS Analysis

#### Chemicals and Materials

Acetonitrile (ACN, 98% purity), methanol (MeOH, 98% purity), pure water (LC–MS grade), ammonium formate (analytical grade) and formic acid (≥ 99% purity) were purchased from VWR International GmbH (Germany). The *S. cerevisiae* α-factor (amino acid sequence: WHWLQLKPQPMY, 1683 g/mol) was ordered from MOLSURF GmbH and Co. KG (Germany). Sulbactam was purchased from Alfa Aesar (USA). Ampicillin (AMP) sodium salt (349 g/mol) was obtained from PanReac AppliChem (Germany) and the AMP isotope-labelled analogue was purchased from Toronto Research Chemicals (Canada). Amoxicillin (AMX) sodium salt (419.4 g/mol), Cloxacilin (CLX) sodium salt (457.46 g/mol), Penicillin G (Pen G) sodium salt (356.37 g/mol) was obtained from Alfa Aesar (USA), Cefalotin (CET) sodium salt (418.42 g/mol) and Piperacillin (PIP) sodium salt (539.54 g/mol) from Sigma Aldrich (USA).

Stock solutions of all antibiotics were prepared separately at a concentration of 1 mg/mL in LC–MS grade water + 0.125% formic acid by weighing the respective solids. The internal standards (ISTD) were dissolved in LC–MS grade water + 0.125% formic acid to a final concentration of 100 mg/L. Sulbactam was dissolved in LC–MS grade water to a concentration of 100 mg/L. The α-factor stock solution was prepared at a concentration of 5 mM. All standards were stored in aliquots at −20 °C.

#### LC and MS Parameters for Analysis of Antibiotics

Analyses were performed using a UHPLC system (Nexera X2 LC-30AD, Shimadzu, Japan) coupled to a QTRAP®6500^+^ tandem mass spectrometer from Sciex (Canada). Analytes were ionized in positive ionization mode via an electrospray ionization (ESI) interface. Evaluation was conducted using MultiQuantTM 3.0.3 software.

*LC-parameters* The chromatographic separation was performed on a TSK gel Amide-80 3 µm 2.0 × 150 mm (TOSOH Bioscience, Japan) column in HILIC mode. It is the same column that allows for α-factor measurement (data not shown). Eluent A consists of 950 mL LC–MS grade water, 50 mL ACN, 10 mM ammonium formate and 1.25 mL formic acid. Eluent B consists of 5 mL LC–MS grade water, 950 mL ACN and 1.25 mL formic acid. The gradient used was 90% eluent B for the initial 3 min, followed by a transition to 10% B from 3.3 min to 5 min, and subsequently reverting to 90% B from 5.5 min to the remaining duration of the analysis (11 min). The flow rate was set at 0.4 mL/min. The temperature of the column oven was set at 40 °C and the sample was maintained at 15 °C within the autosampler. A sample volume of 10 µL was injected.

*MS-parameters* The precursor ion was determined at the maximum intensity depending on the declustering potential (DP), utilizing the integrated optimization programme. Four product ions were automatically determined at the maximum intensity as a function of the collision energy (CE) and collision cell exit potential (CXP), where the two best were used as quantifier and qualifier. The exact parameters can be found in the Supporting Information (SI) in Table S1. In the multiple reaction monitoring (MRM) method the entrance potential (EP) was set to 10 V and the dwell time was set to 50.0 ms. Further defined values are as follows: curtain gas = 40 psi, Gas1 and Gas2 = 50 psi, ion source temperature = 400 °C and IS = 5500 V.

#### Validation

The LC–MS/MS method was validated before measuring the concentration of AMP. Calibration samples were prepared in the concentration range of 1 to 110 nM in LC–MS grade water + 0.125% FA. AMP-ISTD (10 µL) was added to each calibration standard (1000 µL) to a final concentration of 50 nM. The linearity of the method was evaluated by preparing 6 different calibration curves, each freshly prepared, at different times using different batches of reagents. The linearity was determined according to Mandel. The linear approach, defined by the equation y = a·x + b, and the coefficient of determination (R^2^) were employed for regression analysis. The limit of detection (LOD) and limit of quantification (LOQ) were determined in accordance with DIN 32645 using 10 calibration points in at least six replicates within the concentration range of 1 to 110 nM AMP. To assess the intra-day precision, the calibration solutions (10, 50, 100 nM AMP plus a blank) were injected six times within a single day, and the relative standard deviation (RSD) was determined. The recovery percentage was calculated by dividing the measured analyte concentration by the expected concentration. Furthermore, the reproducibility of the method was evaluated by measuring the same samples over an extended period of time and with different reagent batches and operators.

The robustness of the analytical method to practical variations such as column age, mobile phase composition, room temperature and humidity was evaluated by injecting the same calibration curve (AMP plus ISTD) immediately after preparation and after one and eight months. Furthermore, specificity was determined by repeated injection of only water. In order to assess the stability of AMP, AMP solutions were prepared in sulbactam and stored at three different temperatures (4 °C, −20 °C, room temperature) for a period of up to 30 days. Finally, the stability of AMP in water was evaluated through multiple freezing and thawing cycles.

#### Antibiotic Degradation Samples

To verify the degradation of different antibiotics, each antibiotic was considered individually. The supernatant obtained from β-lactamase-producing yeast cultures was vortexed and diluted 1:50 in 0.1 M potassium phosphate buffer (pH 7.4) to a final volume of 350 µL. Subsequently, 70 µL of a solution containing 60 µM of one antibiotic (AMP, AMX, CET, CLX, PEN G or PIP) was added. To monitor the degradation at room temperature over 24 h, 10 µL of the mixture was added to 990 µL of 1 mg/L sulbactam (dissolved in LC–MS grade water) at 0, 10 min, 30 min, 1, 2, 4, 6 and 24 h, respectively, to prevent further degradation. Accordingly, the ultimate dilution ratio was 1:100. Subsequently, 10 µL of 5 µM AMP ISTD was added to the vial, which was then transferred to the autosampler for analysis. To facilitate a comparison of the degradability of the different antibiotics, the same supernatant obtained from TEM1p producing yeasts in W0 medium was used. All experiments were performed in triplicate using independent cultivations.

## Results and Discussion

### Detection of the secreted Enzyme using Western Blot

**Fig. 2:**
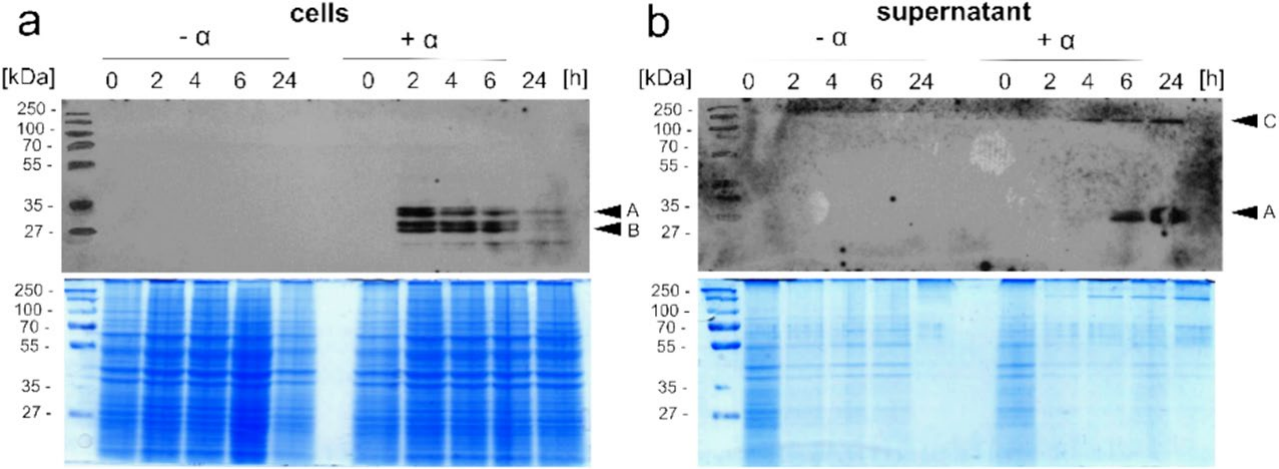
Expression analyses with p426FIG1-HSP150(SS)-TEM1. Yeast was cultivated in W0 medium in the presence of 250 nM α-factor (+ α) or absence (-α) and samples were taken at different time points after induction. Cell extracts (**a**) as well as culture supernatants (**b**) were separated by 12% SDS-PAGE and Western Blot analysis was performed using antibodies against TEM1p (upper panels). Total protein (20 µg per lane) was visualized using colloidal Coomassie staining (lower panels)

To investigate the expression and secretion of TEM1p, samples were collected at specific time points following induction with 250 nM α-factor. Cells (Fig. 2a) as well as culture supernatants (Fig. 2b) were analyzed by SDS-PAGE and Western blot. A control culture, without the addition of α-factor, was run in parallel.

The SDS-PAGE analysis (bottom panels) showed a consistent pattern of protein bands across both cell and supernatant fraction with similar protein loading across the lanes. Western blot analysis was performed using a TEM1p-specific antibody to detect the presence of β-lactamase in the samples (top panels). A band slightly below the 35 kDa marker band was detected in the induced samples, which corresponds to the molecular weight of TEM1p (~ 32 kDa; Fig. 2, arrow A). The bands of lower molecular weight most likely arise from proteolytic degradation products of TEM1p (Fig. 2, arrow B). In the cell fractions, TEM1p can be detected within 2 h of induction. Longer periods of induction result in decreasing levels of TEM1p after 6 and 24 h (Fig. 2a). The decreasing levels of TEM1p in cells is accompanied by increasing levels of TEM1p in the culture supernatant after 6 and 24 h after induction (Fig. 2b). Signals from higher molecular weight species (~ 150 kDa, Fig. 2, arrow C) are visible in the supernatant samples and might represent protein aggregates or complexes. None of the bands can be detected in the control samples (-α) indicating the specificity of the detection. The results of the Western blot analysis confirmed the successful expression and secretion of TEM1p upon induction with α-factor.

### Validation of LC–MS Analysis

Following optimization of the LC–MS/MS parameters (including the column, eluent composition, and gradient—data not shown), linearity was obtained in the concentration range of 1–110 nM AMP with an R-value of 0.99 (see Tab. S2) and was confirmed with the linearity method according to Mandel. A LOD of 2.43 nM and a LOQ of 8.86 nM were calculated (see Tab. S2), which is sufficient for subsequent experiments. Lower LOQs for AMP using LC–MS/MS could be achieved by other investigators by implementing sample pre-treatment procedures, which were not employed in this study, and/or by increasing the injection volume. For instance, Kantiani et al. achieved an AMP LOQ of 22.3 pM in HPLC-grade water on a C12 column using a higher flow rate (0.7 mL/min) and an injection volume of 500 µL [45]. In 2004, Bogialli et al. obtained an LOQ of 5.7 pM after matrix purification (acidification, filtration) using a C18 column with an injection volume of 50 µL [46] and Tlili et al. achieved an LOQ of 0.429 pM after pre-concentration by solid-phase extraction (SPE) and a chromatographic separation on a C18-column [47]. Without sample pre-treatment, Mwankuna et al. achieved a LOQ of 4.37 pM with calibration samples diluted in methanol and water, and Locatelli et al. achieved a LOQ of 1.2 pM in deionized water, both on C18-columns [48, 49].

A high degree of reproducibility was observed (Tab. S3), characterised by a low standard deviation (max. 1.88 nM). The intra-day precision, as expressed in the form of the RSD was 8.8% (for 10 nM AMP), 2.6% (50 nM) and 1.7% (100 nM). The overall recovery was found to be close to 100% (Tab. S4), with the highest recovery rates observed at higher concentrations. The use of ISTD contributes to the robustness of the method (Tab. S5).

Other biochemical or electrochemical methods for differentiating between various β-lactam antibiotics are often inadequate in terms of specificity and sensitivity, as well being laborious and with protracted analysis times [50–52]. In contrast, the LC–MS/MS method presented here exhibited a high degree of specificity within a single run of 11 min.

The addition of the enzyme inhibitor sulbactam at various temperatures, was tested to improve the storage stability of AMP. It is known that sulbactam irreversibly inactivates β-lactamase; β-lactamase has a higher affinity for sulbactam than it does for AMP itself (reviewed in [53]). In the presence of 1 mg/L sulbactam, a loss of about 30% was observed after 14 days at room temperature. At 4 °C, or in a frozen state the AMP concentration remained fairly constant, with losses of less than 10% during the 35-day study period (Fig. S2). Similar observations, such as a higher degradation rate at higher temperatures, have been previously documented by Savello & Shangraw in pure water [54]. However, stability is not solely contingent on temperature, it is also influenced by the solvent and concentration, as previously documented [54–58].

Additionally, the effect of multiple cycles of freezing and thawing in LC–MS grade water was investigated (Fig. S3) to account for potential losses in the stock solutions. The concentration remained stable after 5 freeze–thaw cycles and even on day 35 (following 11 freeze–thaw cycles) the residual AMP concentration was 75% of the starting level. In contrast, Holmes et al. observed a loss of stability during the freeze–thaw cycle, even after the initial run, when AMP was dissolved in pure water with 5% dextrose [59].

### AMP Degradation using LC–MS Analysis and Nitrocefin Assay

#### Influence of the Cultivation Time

**Fig. 3:**
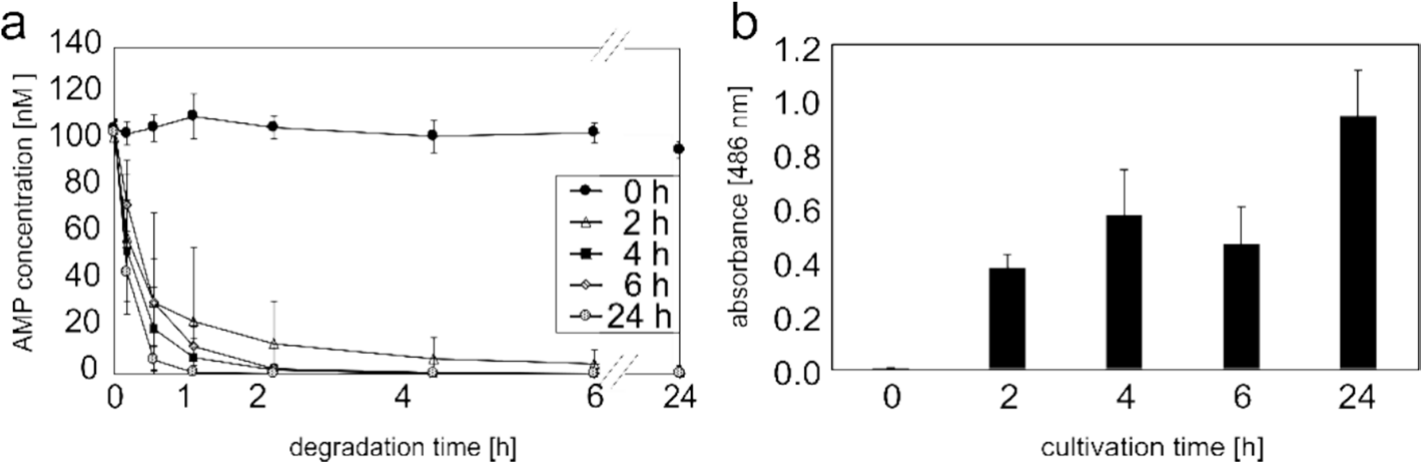
Influence of cultivation period on the presence of β-lactamase in the supernatant. Detection of β-lactamase content was performed indirectly through the degradation of 10 µM AMP—analyzed by (**a**) LC–MS/MS and (**b**) measurement of absorbance at 486 nm in the Nitrocefin assay. *S. cerevisiae* was cultivated in W0-medium. Data represent mean values ± standard deviation from three independent experiments

AMP degradation was analyzed in the cell culture supernatant to verify that β-lactamase TEM1p was secreted to the outside of the yeast cell. As illustrated in Fig. 2, the Western blot analysis revealed the presence of β-lactamase TEM1p in the supernatant after 6 and 24 h of cultivation, indicating an increased cell response with increasing cell density. Conversely, this should result in an increase in AMP degradation over time. Therefore, the β-lactamase secretion associated with yeast growth in the culture medium was indirectly detected by AMP degradation using LC–MS/MS (Fig. 3a) and, for comparison, by the nitrocefin assay (Fig. 3b). Therefore, a relatively high concentration of α-factor (250 nM) was introduced into the cultivation media at time zero. Following 0, 2, 6 and 24 h of cultivation (= cultivation period), a sample was taken and the supernatant was separated from the yeast (solid, pellet material). The supernatant was used to examine the degradation of AMP over a 24-h period (= degradation period; Fig. 3a) or to indirectly ascertain the quantity of enzyme at designated time points (Fig. 3b).

In supernatant samples collected at the beginning of the cultivation stage (time zero; Fig. 3a), no degradation of AMP was observed over the course of 24 h. Only minimal differences (9%) between the initial and final AMP concentrations in the time zero sample were observed. These differences could be attributed to chemical hydrolysis. However, the longer the yeast is cultured with the inducing α-factor, the faster the degradation occurs. Note that the 4 and 6 h samples are largely indistinguishable. This apparent stagnation can be attributed to the fact that α-factor is used to regulate the cell cycle. So, the lack of increase in activity is also reflected in the cell density of *S. cerevisiae* (a minimal increase in optical density measurements between 4 and 6 h of cultivation is observed; Tab. S6). Nevertheless, in samples taken following 4 or 6 h cultivation periods, sufficient TEM1p had been produced to degrade over 90% of the AMP within 1 h. This same milestone was achieved within 0.5 h in the supernatant sample taken after a 24 h cultivation period. The increasing degradation over time, resulting in a higher absorbance corresponding to higher TEM1p content (and the apparent stagnation in the 4 to 6 h range) were also confirmed by the Nitrocefin assay (Fig. 3b), which is not as specific, and even not as sensitive, as the LC–MS/MS reported here. The effects of growth-dependent production and the higher secretion rate due to higher cell density corroborate earlier findings [60, 61].

#### Influence of α-factor Concentration

**Fig. 4:**
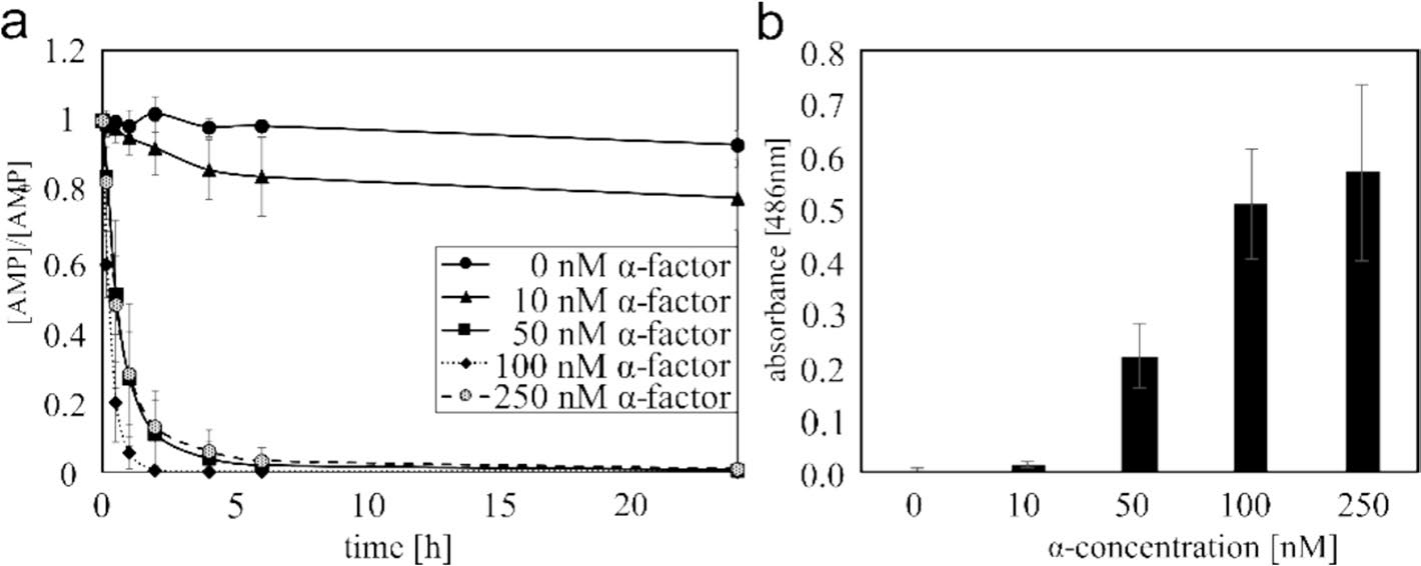
Influence of different α-factor concentrations on the secretion of β-lactamase in the supernatant of *S. cerevisiae* cultures in W0 medium following 4 h of cultivation. Expression of *TEM1* in yeast was induced using the α-factor concentrations indicated in the inset. Concentration of AMP was measured (**a**) indirectly by analysing the degradation of 10 µM AMP over a 24-h period in the 1:50 diluted supernatant. The values obtained from three different yeast cultivations and are normalized to c_0._ (**b**) Further analyses of the same supernatant were carried out using the nitrocefin assay. The supernatant was diluted 1:5 before starting the experiment. The blank (media) was subtracted. Data represent mean values ± standard deviation (*n* = 3)

The impact of α-factor, an unmodified peptide comprising 13 amino acids, on the AMP degradation rate was evaluated by introducing varying concentrations of α-factor to shaking flasks containing *S. cerevisiae*. Due to the high AMP degradation rate observed following a 24 h of cultivation period (data not shown), the influence of distinct α-factor concentrations was further examined using supernatants extracted after a cultivation period of 4 h. Figure 4a shows the degradation of 10 µM AMP in yeast culture supernatants over a period of 24 h – displayed according to the α-factor concentration used during the 4 h cultivation period. Figure 4b presents the results of the nitrocefin assay after 4 h of cultivation with varying concentrations of α-factor.

Here, for yeast cultivated without the addition of α-factor, only a minor decrease in AMP concentration was observed. This slight decrease may be attributed to non-enzymatic processes such as hydrolysis or interactions with media components, as previously observed by Ramírez Muñoz [62]. In the presence of 10 nM α-factor, 30% of the AMP can be degraded within 24 h. After 24 h incubation, almost all AMP was degraded when using α-factor concentrations of 50 nM and above. Use of 100 nM α-factor and a subsequent 1:50 dilution of the supernatant results in almost 96% degradation of 10 µM AMP (3.5 mg/L) within 1 h. In conclusion, the steady rate of production of β-lactamase TEM1p, due to the high concentration of inducer (α-factor) enhanced the reaction rate and degradation. However, it should be noted that higher α-factor concentrations are associated with lower cell densities (Tab. S6). Optical densities were recorded as being almost identical during the first 2 h, regardless of α-factor concentration (≤ 5%). However, after 4 h in the presence of 50 nM α-factor, the optical density was only at 75% of the sample without α-factor (Tab. S6). The stagnation of cell growth was greater with increasing α-factor concentration, as the α-factor influenced the cell cycle arrest (reviewed in [63]). This phenomenon has also been observed by Hennig et al. for *Schizosaccharomyces pombe* with a 4 h arrest before growth occurs [60]. Similarly, Zanolari et al. reported a stagnation period of 2.5 h [64]. The arrest time depends on the number of cells, as well as the cell medium and the age of the cells [65]. The observed increase in degradation (enzymatic hydrolysis of AMP) despite the low increase in cell density with increasing α-factor can be attributed to the FIG1 promoter, which induces the heterologous expression of TEM1p after α-factor is recognized by the cells. This phenomenon has been previously described by Yashiroda and Yoshida and Hennig et al. in their studies [60, 66]. In general, the presence of α-factor results in the production of a greater quantity of enzyme and thus the enzymatic hydrolysis of AMP is accelerated.

In comparison to established methods, such as granular activated carbon which requires 2 h to remove 73% of 200 mg/L AMP [16], or Cu(II)-catalysed hydrolysis, which requires 4 h to degrade 85.9% of 35 mg/L AMP [67] or photocatalytic processes, where 3.8% of 105 mg/L AMP remains after 5 h of UV irradiation [26], the use of bioengineered yeast represents an alternative for the degradation of even low concentrations of AMP. Only 3.7% of the initial 3.5 mg/L AMP remaining after 1 h, achieved using only 100 nM α-factor and a 1:50 dilution of the supernatant. Additionally, in comparison to physico-chemical processes, the generation of toxic by-products with the necessity for subsequent separation and disposal is avoided, as well as the requirement for significant energy inputs.

### Formation of AMP Transformation Products

**Fig. 5:**
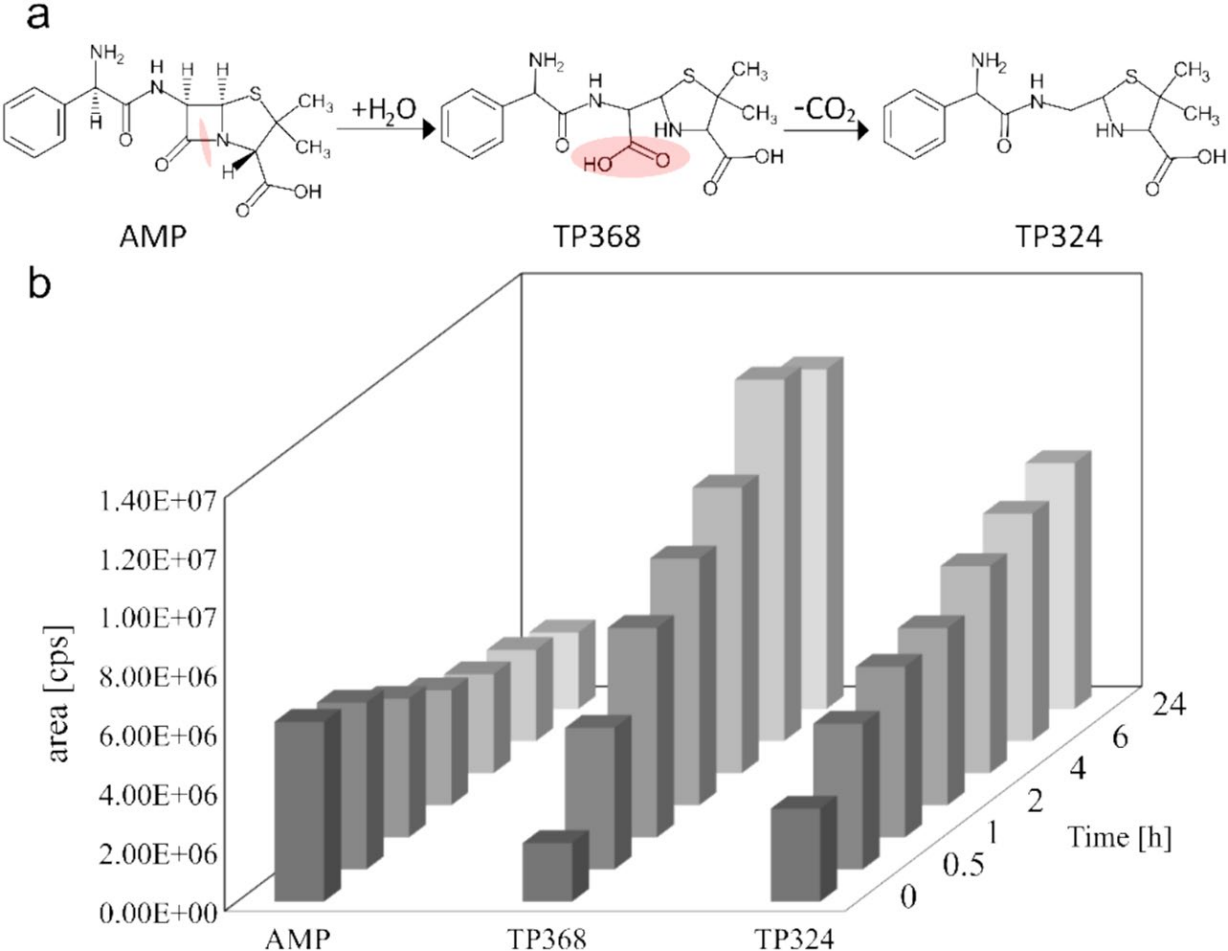
Formation of transformation products from AMP (**a**) Pathway for the generation of transformation products m/z = 368 and m/z = 324 from AMP—cleavage sites marked and (**b**) TP formation, measured in the form of detected peak areas for AMP, TP368 and TP324; analysis conducted over a degradation period of 24 h. *S. cerevisiae* was cultivated for 24 h in W0 medium. The supernatant was harvested, diluted 1:50 and then used in the degradation study for TP analysis. AMP and the transformation products m/z = 368 and m/z = 324 were detected

Evidence of the degradation of AMP was provided by the determination of transformation products (TPs). TPs can be formed through a range of processes, including oxidation, biodegradation, hydrolysis, and even photolysis (reviewed in [68, 69]). In this study, AMP is observed to be close to completely stable over the experimental period of 24 h (Figs. 3 and 4). Therefore, TP formation by chemical hydrolysis and photolysis is considered to be negligible, and thus degradation is attributed exclusively to enzymatic activity. The cleavage of the β-lactam ring results in the loss of the antibiotics'efficacy (reported in [35]). A variety of TPs can be (temporarily) detected during the degradation of AMP [70]. According to Dumancas et al., the resulting TPs are less toxic than the parental compound [35] but they are relatively persistent compared to the parental compound [71]. The toxicity of AMP was investigated by Lalas et al. under AOP conditions, a process which is known for the formation of toxic by-products. The toxicity of AMP and some TPs was compared resulting in a not increasing toxicity, which is why it is assumed that no toxic by-products are formed [72]. Furthermore, the AMP TPs formed in the study of Raiker et al. were predicted to be non-toxic by using the ECOSAR model [73]. Raiker et al. also investigated the two major TPs of AMP, namely TP368 (= penicilloic acid) and TP324 (= penilloic acid). These TPs are not harmful with respect to the acute toxicity and TP324 is only slightly harmful concerning chronic toxicity in daphnids [73]. Furthermore, within the UVC LED/PS method he used, the antibiotic activity is lost and no by-products with antibacterial activity are produced [73]. Furthermore, a comparison with AMX, which forms similar TPs (AMX penicilloic acid and AMX penilloic acid), shows that aerobic composting leads to less toxic products compared to the starting products, which also appear to be non-allergenic [74]. Finally, a targeted analysis of the two major typical enzymatic hydrolysis TPs of AMP (TP368 and TP324), was performed to verify the enzymatic turnover. Figure 5a illustrates the pathway from AMP to TP368 and TP324, and Fig. 5b the corresponding peak areas detected. Further fragments were qualitatively classified through product ion scans, as illustrated in Fig. S4 and S5 in the SI.

The formation of both TP368 and TP324 typically occurs following cleavage of the β-lactam ring and the addition of water to the AMP molecule, resulting in the formation of TP368 (penicilloic acid). Subsequent cleavage of the acid group leads to the formation of TP324 (penilloic acid; Fig. 5a).

The presence of both TPs at the time zero point may be attributed to partial hydrolysis of AMP during storage in LC–MS grade water + 0.125% FA (given that the AMP stock solution was not freshly prepared; Fig. 5b). The presence of FA facilitates hydrolysis and resulting in the formation of the same products as generated by enzymatic degradation [75]. The peak areas show a decrease in AMP concentration and an increase in TP over the degradation period. In the initial stages, TP368 formed in increasing quantities, a phenomenon that was also documented by Arsand et al. [70]. Subsequently, TP368 underwent spontaneous acid decarboxylation, a process that had previously been identified by Suwanrumpha & Freast [76]. Consequently, the area of TP368 decreases slightly at 24 h, while that of TP324 continues to increase with time. It should also be noted that an elevated peak area for m/z = 324 can be achieved through the degradation of another intermediate product of AMP [76]. Furthermore, more transformation products can be formed out of TP368 and TP324, as Suwanrumpha & Freast mentioned [76].

### Transferability of AMP degradation to other β-lactam antibiotics

**Fig. 6:**
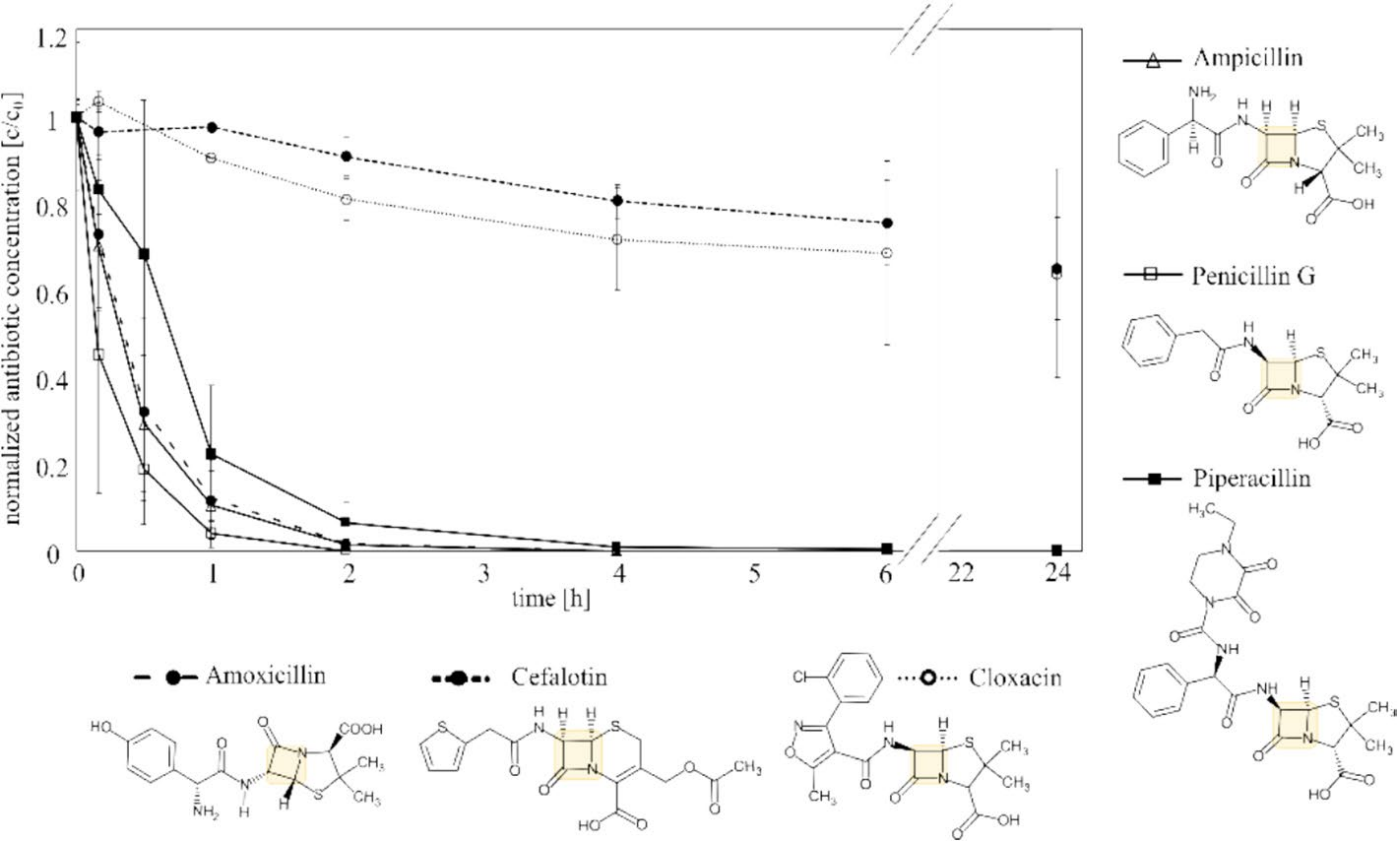
Degradability of different antibiotics using supernatant containing TEM1p. Prior to beginning the timecourse, *S. cerevisiae* was cultivated in W0 medium containing 250 nM α-factor for 24 h. The supernatant obtained after 24 h was diluted 1:50, and 10 µM AMP, AMX, CLX, CEL, PEN G or PIP (in separate vials) were added to analyze degradability over a period of 24 h. Data represent mean values ± standard deviation for the three independent experiments. Furthermore, structural formulae of Ampicillin (AMP), Amoxicillin (AMX), Penicillin G (PEN G), Piperacillin (PIP), Cloxacillin (CLX) and Cefalotin (CET) is shown. The β-lactam ring is highlighted in yellow

Thus far, the degradation activity of the β-lactamase TEM1p has only been demonstrated against AMP, a penicillin with a broad spectrum of action. However, the β-lactam antibiotic group also encompasses cephalosporins, carbapenems and monobactams which exhibit differences in terms of their spectrum of activity and pharmacokinetic profiles, with the R1 side chain being partially responsible for these different behaviours [77, 78]. It is interesting to study whether the TEM1p produced in this study is as effective at degrading the commonly prescribed AMX, PEN G and PIP (as penicillins [79]), as well as CLX (a penicillase resistant penicillin), and also CET (a first generation cephalosporin with activity against gram positive bacteria). In terms of their structural composition (Fig. 6), all of the aforementioned antibiotics contain the specific β-lactam ring that is recognised by the β-lactamase.

The β-lactamase enzyme cleaves the four-membered lactam ring to hydrolyse the amide bond, resulting in inactive antibiotics (see review [80]). The orientation of the carbonyl and carboxylate side groups are relevant for the lactamase activity [81]. However, all penicillins contain a dihydrothiazine ring, whereas cephalosporins (e. g. CLT) possess a thiazolidine ring. Additionally, all penicillins contain a benzene ring. Piperacillin contains an additional C6 ring, which could result in a slower degradation process. CLX contains a heterocyclic compound and an additional chlorine atom, which could impede its degradation. In contrast, CLT contains a thiophene, which could account for its inferior stability.

Therefore, the degradability of these β-lactam antibiotics was tested in the supernatant of *S. cerevisiae* containing the secreted TEM1p. The results are presented in Fig. 6.

The degradation of all lactams tested was observed over a period of 24 h. CLX, (a penicillinase-resistant penicillin derivative) and CLT (a cephalosporin) were only degraded by approximately 30%. Meanwhile, the other four antibiotics were almost completely degraded within 24 h. The β-lactamase sensitive antibiotic PEN G exhibited the fastest rate of degradation, followed by the aminopenicillins AMP and AMX, and the acylaminopenicillin PIP. The concentration of Cefadroxil, also a first-generation cephalosporin was constant over the time (data not shown), despite being structural similar to AMX. As previously demonstrated by Cantu et al., the specificity of TEM1p for penicillin (AMP and PEN-G) is higher than for the cephalosporin (CLT) they tested [82]. Furthermore, Farrar and O’Dell provided additional evidence for the enhanced stability of certain cephalosporins compared to penicillin [83]. Additionally, cephalosporins have been employed as an alternative to penicillin in some clinical studies, with some patients requiring a 5–20 times higher dose to achieve the same results [78].

The varying efficiency of degradability depends on several factors, including the sensitivity and affinity of the antibiotics for the enzyme in question, as well as the quantity of enzyme present [84]. Here, the supernatant was diluted at a 1:50 ratio. However, a complete degradation of CLX and CLT may occur in the absence of dilution, with a more rapid degradation of the other tested antibiotics. Consequently, the method is a promising approach to accelerate the degradation of various antibiotics in wastewater.

## Conclusion

The widespread consumption of β-lactam antibiotics, coupled with their insufficient removal in WWTPs, has led to environmental concentrations in the ng/L-µg/L range, which, in turn, favours the development of antibiotic resistance. Consequently, a robust and effective methodology for the degradation of β-lactam antibiotics is imperative.

In this study, a novel system for the inducible secretion of β-lactamase, an enzyme for degrading β-lactam antibiotics by hydrolysing the lactam ring, by a yeast as a part of a cross-kingdom communication system, was presented. Therefore, *S. cerevisiae* was genetically modified by the vector-based integration of the β-lactamase gene *TEM1*, which is fused at its N-terminal to the coding region of the secretion signal of *HSP150*. The resulting construct is placed under the control of the FIG1 promoter. In the presence of α-factor, the FIG1 promoter is activated and *S. cerevisiae* starts to produce and secrete the enzyme β-lactamase (TEM1p). This modification was demonstrated by Western Blot analysis, where only TEM1p was detected in the supernatant. The ability of the enzymes to degrade AMP as a model substrate was also tested. For this propose an analytical method was established that employs HILIC separation on a TSKgel column for a fast, robust and sensitive detection and quantification of AMP with a LOD of 2.43 ± 0.56 nM and LOQ of 8.86 ± 1.94 nM. The kinetics of AMP degradation depended on both cell density of yeast and α-factor concentration, both of which led to an increase in secreted β-lactamase concentration.

The β-lactamase TEM1p produced here can be also used for the degradation of other β-lactam antibiotics. Amoxicillin (AMX), Penicillin G (PEN G), Piperacillin (PIP) showed similar degradation rates to AMP, while as expected Cloxacillin (CLX) and Cefalotin (CET) were more stable.

In the future, the modified yeast will be integrated into a sensor-actuator system in which the formation of antibiotic-degrading enzymes is induced by signals (α-factor) generated in sensor cells upon sensing antibiotics in wastewater. The controllability of expression and the successful degradation of β-lactam antibiotics by the secreted enzyme (β-lactamase) have already been demonstrated in the present work. The enzymes produced can be used in WWTPs as an alternative to chemical and/or sorption techniques. Furthermore, immobilization of the enzymes could be considered, given that the degradation of other pharmaceuticals is already a well-established practice (review [85]). In addition to antibiotics, the yeast system could also be engineered to produce enzymes for the degradation of recalcitrant substances or other classes of pollutants. Currently, the modified yeast is capable of producing enzymes such as lipases or even peroxidases and oxidases for the degradation of aromatic substances [86].

## Supplementary Information

Below is the link to the electronic supplementary material.

ESM 1 (DOC 3.49 MB)

## Data Availability

The data is available in the main manuscript and in the supplementary file if mentioned**.**
