# Supplementary material for: Genetically Engineered Yeast for Enhanced Biodegradation of Β-lactam Antibiotics
Source: Appl Biochem Biotechnol. 2025 Jun 27;197(9):5649–67. doi: 10.1007/s12010-025-05291-4 (PMC12568803; doi:10.1007/s12010-025-05291-4)
Supplement: Supplementary file 1 — (DOC 3.49 MB) [file 12010_2025_5291_MOESM1_ESM.doc]

Supplementary Information

**Genetically engineered Yeast for enhanced Biodegradation of β-lactam Antibiotics**

Carolin Pohl^1[0009-0009-1150-3133]^, Cindy Rau^1,2^, Linda Schuster^1^, Uta Gutbier^3,4^, Stephan Beil^1^, Katrin Lehmann^4^, Hilmar Börnick^1^, Kai Ostermann^4^ and Stefan Stolte^1^[0000-0001-5186-3955]

^1^ Faculty of Environmental Science, Institute of Water ChemistryTUD Dresden University of Technology, 01062 Dresden, Germany

^2^ Faculty of Civil Engineering, Water Engineering, HTW Hochschule für Technik und Wirtschaft, Friedrich-List-Platz 1, 01069 Dresden, Germany

^3^ Else Kröner Fresenius Center for Digital Health, Faculty of Medicine Carl Gustav Carus, TUD Dresden University of Technology, Dresden, Germany

^4^ Faculty of Biology, Research Group Biological Sensor-Actuator-Systems, TUD Dresden University of Technology, 01062 Dresden, Germany
stefan.stolte@tu-dresden.de

**Fig. S1** HSP150 (SS) – *TEM1* (codon optimized nucleotide sequence)

ATG CAA TAC AAA AAG ACT TTG GTT GCC TCT GCT TTG GCC GCT ACT ACA TTG GCC GCC TAT GCT CCA TCT GAG CCT TGG TCC ACT TTG ACT CCA ACA GCC ACT TAC AGC GGT GGT GTT ACC GAC TAC GCT TCC ACC TTC GGT ATT GCC GTT CAA CCA ATC TCC ACT ACA TCC AGC GCA TCA TCT GCA GCC ACC ACA GCC TCA TCT AAG GCC AAG AGA GCT GCT TCC CAA ATT GGT GAT GGT CAA GTC CAA GCT GCT ACC ACT ACT GCT TCT GTC TCT ACC AAG AGT ACC GCT GCC GCC GTT TCT CAG ATC GGT GAT GGT CAA ATC CAA GCT ACT ACT AAG ACT ACC GCT GCT GCT GTC TCT CAA ATT GGT GAT GGT CAA ATT CAA GCT ACC ACC AAG ACT ACC TCT GCT AAG ACT ACC GCC GCT GCC GTT TCT CAA ATC AGT GAT GGT CAA ATC CAA GCT ACC ACC ACT ACT TTA GCC CCA AAG AGC ACC GCT GCT GCC GTT TCT CAA ATC GGT GAT GGT CAA GTT CAA GCT ACC ACC ACT ACT TTA GCC CCA AAG AGC ACC GCT GCT GCC GTT TCT CAA ATC GGT GAT GGT CAA GTT CAA GCT ACT ACT AAG ACT ACC GCT GCT GCT GTC TCT CAA ATT GGT GAT GGT CAA GTT CAA GCT ACC ACC AAG ACT ACT GCT GCC GCC GTT TCT CAA ATC GGT GAT GGT CAA GTT CAA GCT ACT ACC AAG ACT ACC GCT GCT GCT GTC TCT CAA ATC GGT GAT GGT CAA GTT CAA GCA ACT ACC AAA ACC ACT GCC GCA GCT GTT TCC CAA ATT ACT GAC GGT CAA GTT CAA GCC ACT ACA AAA ACC ACT CAA GCA GCC AGC CAA GTA AGC GAT GGC CAA GTC CAA GCT ACT ACT GCT ACT TCC GCT TCT GCA GCC GCT ACC TCC ACT GAC CCA GTC GAT GCT GTC TCC TGT AAG ACT TCT GGT ACC TGC AGT CAT CCG GAA ACC TTA GTC AAA GTG AAA GAT GCG GAG GAC CAA TTG GGT GCT AGA GTT GGA TAT ATT GAA TTG GAT CTC AAC TCA GGC AAG ATT CTG GAA TCC TTT CGT CCT GAG GAA CGA TTT CCT ATG ATG TCT ACC TTC AAA GTG TTA CTC TGC GGA GCC GTA CTG TCT AGA GTT GAC GCG GGT CAG GAA CAA CTA GGT AGA CGC ATA CAC TAC AGT CAG AAT GAT CTA GTT GAA TAC AGC CCA GTT ACT GAG AAG CAT CTT ACT GAT GGC ATG ACA GTC AGA GAA CTG TGT AGC GCA GCC ATT ACT ATG TCG GAT AAT ACA GCT GCC AAC TTA CTG TTG ACT ACC ATT GGT GGA CCC AAA GAG TTG ACA GCT TTT CTA CAC AAC ATG GGT GAC CAT GTC ACT AGG TTG GAT AGG TGG GAA CCA GAA CTC AAT GAA GCT ATT CCC AAT GAC GAA CGT GAT ACG ACG ATG CCA GCT GCC ATG GCT ACC ACA CTA CGC AAG TTA CTT ACT GGT GAA CTT CTG ACA CTT GCC TCT AGA CAA CAG TTG ATT GAT TGG ATG GAA GCA GAT AAA GTA GCA GGA CCT CTA TTA CGT AGT GCC TTG CCA GCT GGG TGG TTC ATA GCA GAT AAG AGT GGA GCA GGT GAA CGA GGG TCA AGA GGC ATC ATT GCA GCT TTA GGT CCA GAC GGC AAA CCG TCA CGG ATA GTA GTT ATC TAT ACT ACA GGC TCT CAA GCG ACT ATG GAT GAA AGG AAT AGG CAA ATT GCA GAG ATT GGT GCT TCC TTG ATC AAA CAT TGG TAA

**Table S1** LC and MS parameters

| Compound | Molecular formular | Ret. time (min) | Q1 (m/z) | Q2 (m/z) | CE (V) | DP (V) | CXP (V) |
| --- | --- | --- | --- | --- | --- | --- | --- |
| Ampicillin | C_16_H_19_N_3_O_4_S | 4.53 | 350 | 106 | 21 | 41 | 20 |
| Ampicillin ISTD | C_16_H_14_D_5_N_3_O_4_S | 4.53 | 355 | 111 | 21 | 66 | 12 |
| Cefadroxil | C_16_H_17_N_3_O_5_S | 4.67 | 364 | 208 | 13 | 6 | 24 |
| Amoxicillin | C_16_H_19_N_3_O_5_S | 4.85 | 366 | 349 | 11 | 5 | 18 |
| Cefalotin | C_16_H_16_N_2_O_6_S_2_ | 1.05 | 397 | 337 | 15 | 46 | 18 |
| Cloxacilin | C_19_H_18_ClN_3_O_5_S | 1.00 | 435 | 277 | 21 | 41 | 32 |
| Penicillin G | C_16_H_18_N_2_O_4_S | 1.03 | 335 | 176 | 17 | 41 | 20 |
| Piperacillin | C_23_H_27_N_5_O_7_S | 1.05 | 518 | 143 | 21 | 46 | 16 |

**Table S2** Overview of calibration curves with different batches of reagents in a concentration range of 1 – 110 nM

|  | Y=a·x + b | R | LOD (nM) | LOQ (nM) |
| --- | --- | --- | --- | --- |
| 1 | Y = 0.971x + 0.965 | 0.9992 | 2.36 | 8.5 |
| 2 | Y = 0.993x + 0.938 | 0.999 | 3.05 | 11.1 |
| 3 | Y = 1.002x + 0.509 | 0.9986 | 3.2 | 11.5 |
| 4 | Y = 0.992x - 0.938 | 0.9996 | 1.5 | 5.8 |
| 5 | Y = 0.977x +0.628 | 0.9992 | 2.30 | 8.36 |
| 6 | Y = 1.04x -1.023 | 0.9993 | 2.16 | 7.889 |
| Mean value and standard deviation | | | **2.43 ± 0.56** | **8.86 ± 1.94** |

**Table S3 Determination of the reproducibility of the analytical method through the preparation of two different calibration curves by different operators**

| Reproducibility | 1. | 2. | Mean value | Standard  deviation |
| --- | --- | --- | --- | --- |
| 10 | 10.55 | 9.97 | 10.26 | 0.29 |
| 20 | 20.98 | 19.01 | 19.995 | 0.985 |
| 30 | 31.09 | 28.81 | 29.95 | 1.14 |
| 40 | 40.14 | 40.16 | 40.15 | 0.01 |
| 50 | 48.52 | 50.64 | 49.58 | 1.06 |
| 60 | 59.86 | 63.62 | 61.74 | 1.88 |
| 70 | 69.99 | 69.52 | 69.755 | 0.235 |
| 80 | 80.22 | 82.55 | 81.385 | 1.165 |
| 90 | 90.98 | 89.85 | 90.415 | 0.565 |
| 100 | 98.03 | 98.49 | 98.26 | 0.23 |
| 110 | 110.9 | 108.4 | 109.65 | 1.25 |
| Y = ax + b | Y =  0.992x + 0.5918 | Y =  0.9971x + 0.2645 |  |  |
| R-value | 0.9991 | 0.9975 |  |  |

**Table S4 Repeatability of the analytic method used. Determination through seven injections of three different concentrations every 35 min**

| Repeatability | 10 | 50 | 100 |
| --- | --- | --- | --- |
| 1 | 11.37 | 47.96 | 99.48 |
| 2 | 9.83 | 50.21 | 98.78 |
| 3 | 10.36 | 49.87 | 97.58 |
| 4 | 10.17 | 50.52 | 102.79 |
| 5 | 9.67 | 51.76 | 100.79 |
| 6 | 10.55 | 49.28 | 100.83 |
| 7 | 12.57 | 47.92 | 102.22 |
| Mean value and standard deviation | 10.65 ± 0.94 nM | 49.65 ± 1.28 nM | 100.35 ± 1.72 nM |
| Ø Recovery | 106 % | 99 % | 100 % |
| RSD | 8.8 % | 2.6 % | 1.7 % |

**Table S5 Robustness of the analytical method by re-injection of the calibration curve after one month and again after eight months**

| Concentration of AMP (nM) | Calculated concentration (fresh) | Calculated concentration (after one month) | Calculatedconcentration (after 8 month) | Mean value | Standard deviation |
| --- | --- | --- | --- | --- | --- |
| 1 | 2.77 | 2.47 | 4.85 | 3.36 | 1.06 |
| 5 | 6.94 | 6.74 | 7.30 | 6.99 | 0.23 |
| 10 | 9.94 | 10.23 | 10.44 | 10.20 | 0.21 |
| 20 | 19.57 | 19.31 | 19.37 | 19.41 | 0.11 |
| 30 | 28.72 | 29.72 | 30.47 | 29.63 | 0.72 |
| 40 | 38.78 | 40.70 | 39.17 | 39.55 | 0.83 |
| 50 | 48.75 | 49.76 | 46.06 | 48.19 | 1.56 |
| 60 | 58.02 | 60.09 | 58.88 | 59.00 | 0.85 |
| 70 | 68.44 | 65.17 | 65.73 | 66.45 | 1.43 |
| 80 | 81.06 | 74.88 | 73.16 | 76.37 | 3.39 |
| 90 | 87.86 | 92.65 | 88.15 | 89.55 | 2.20 |
| 100 | 102.51 | 101.33 | 105.03 | 102.96 | 1.54 |

**
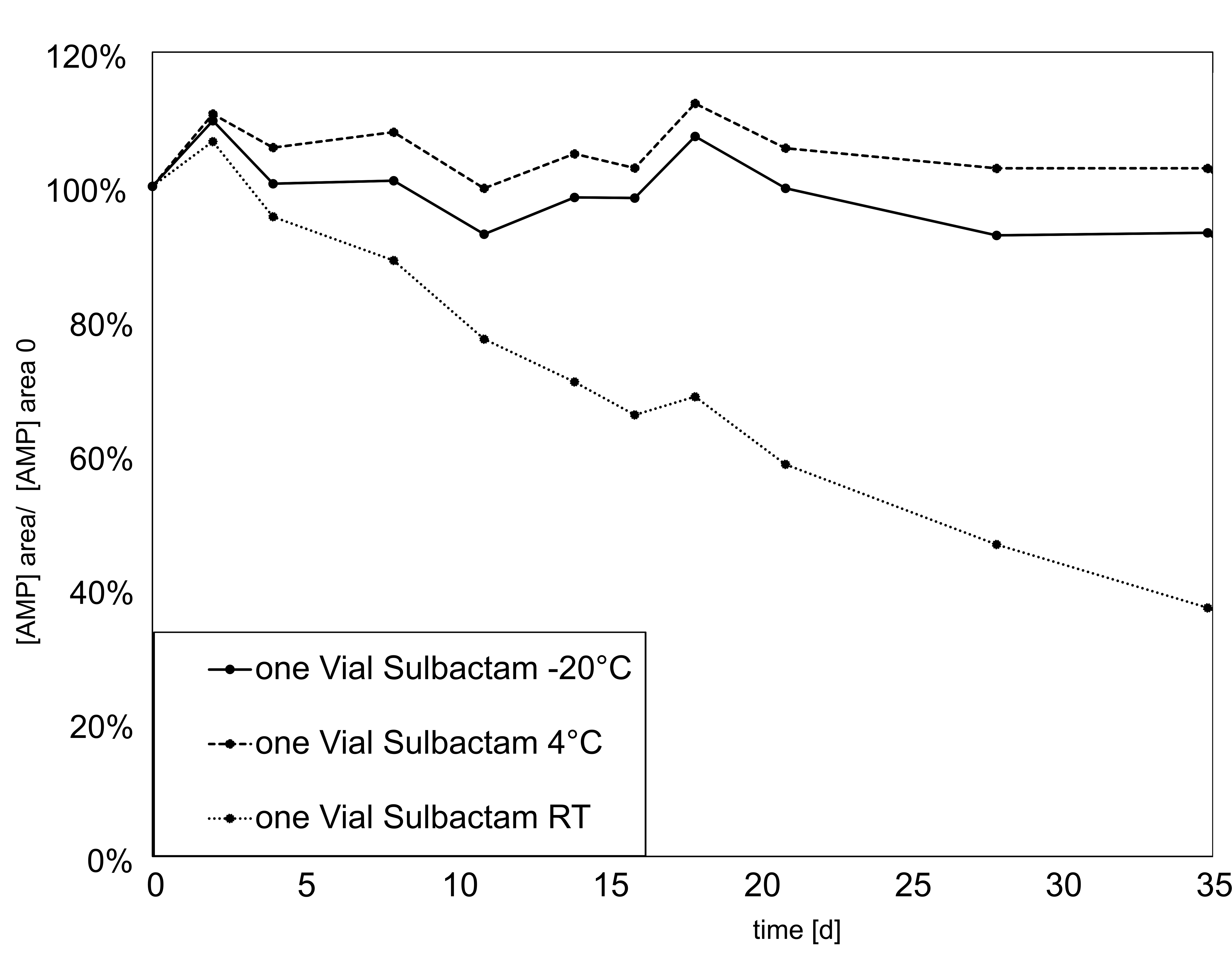
**

**Fig. S2** Investigation of the stability of AMP in the presence of Sulbactam without the use of an internal standard. The AMP-values were obtained over a period of 35 days with repeated freeze/ thaw cycles or even warming or cooling to the temperature of the autosampler. AMP was stored at -20°C, 4°C and at room temperature (n=1)


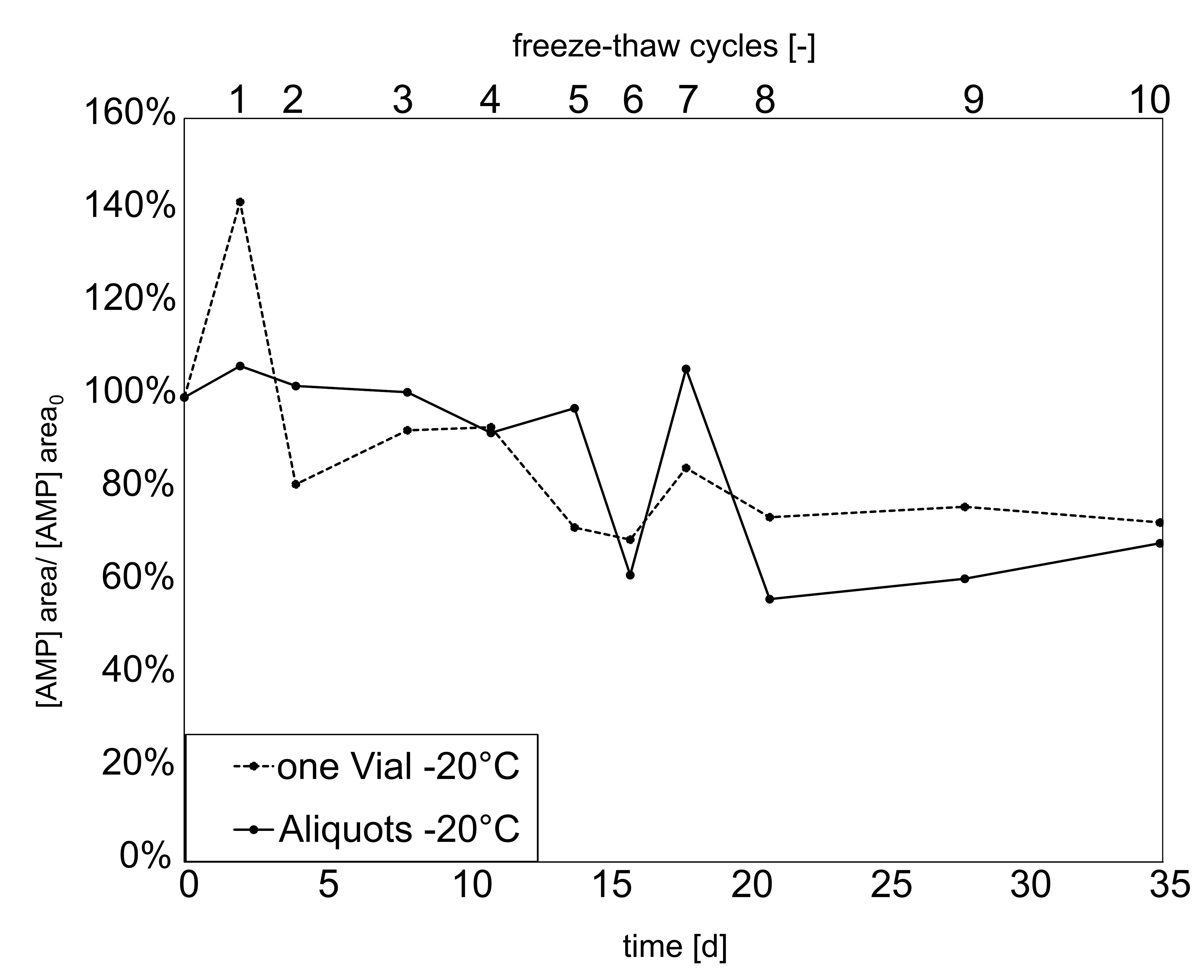


**Fig. S3** Comparison of AMP storage at -20°C. Analyzing and comparing the peak area when taking samples from Aliquots or taking the sample from one vial over a period of 35 days. No internal standard was used (n = 1)

**Table S6** Optical densities of the three cultures of *S. cerevisiae* with and without the addition of α-factor at the indicated concentrations (0, 10, 50 and 250 nM)

|  | experiment 1 | | | | | experiment 2 | | | | | experiment 3 | | | | |
| --- | --- | --- | --- | --- | --- | --- | --- | --- | --- | --- | --- | --- | --- | --- | --- |
| α [nM]  Time | 0 | 10 | 50 | 100 | 250 | 0 | 10 | 50 | 100 | 250 | 0 | 10 | 50 | 100 | 250 |
| 0 h | 1.03 | 0.98 | 1.02 | 1.01 | 0.98 | 1.02 | 1.00 | 1.00 | 0.99 | 1.00 | 1.01 | 1.02 | 1.01 | 1.03 | 1.02 |
| 2 h | 1.28 | 1.29 | 1.34 | 1.34 | 1.34 | 1.55 | 1.42 | 1.45 | 1.43 | 1.42 | 1.48 | 1.42 | 1.48 | 1.35 | 1.42 |
| 4 h | 2.02 | 1.70 | 1.70 | 1.60 | 1.57 | 2.63 | 2.37 | 2.00 | 1.88 | 2.37 | 2.64 | 2.58 | 1.80 | 1.84 | 2.58 |
| 6 h | 3.27 | 2.79 | 2.08 | 1.92 | 1.83 | 3.10 | 2.85 | 2.34 | 2.24 | 2.85 | 3.06 | 2.90 | 2.44 | 2.46 | 2.90 |
| 24 h | 3.78 | 3.69 | 3.20 | 3.03 | 2.78 | 4.28 | 3.98 | 3.68 | 3.54 | 3.98 | 4.00 | 4.55 | 3.25 | 3.60 | 4.55 |

### Transformation Products

Penicilloic acid (TP368) is the more intense TP and is also formed by photolysis and hydrolysis (Arsand et al.. 2018). For this reason, only this will be considered for determining the fragments. The fragments were only classified qualitatively through product-ion-scans where a range of the predicted TPs were selected and the fragments determined. Possible fragments of TP 368 are shown in Figure S4. The fragments that are above the average intensity (blue dotted line) are considered more closely.


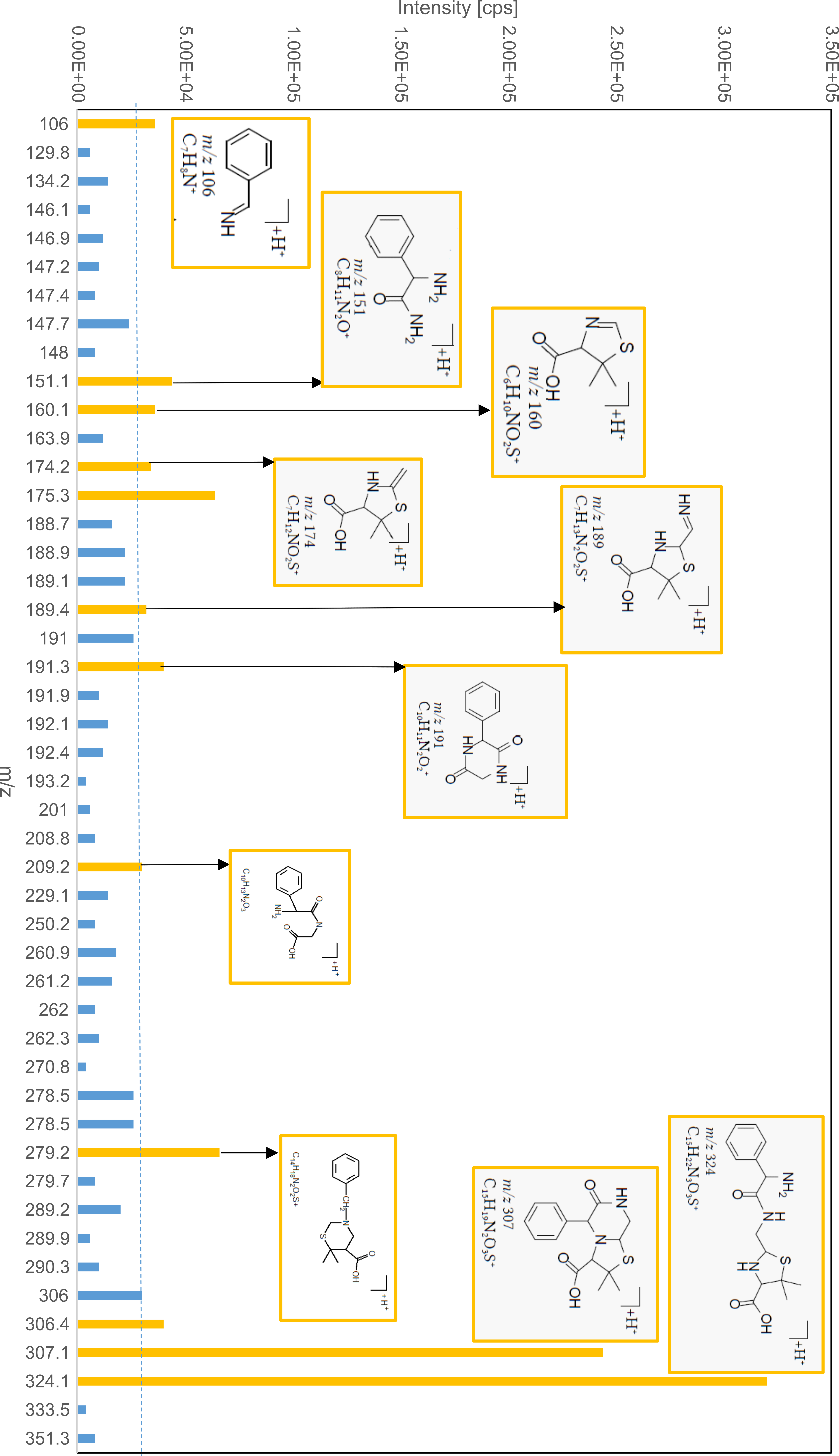


**Fig. S4** Determined fragments from TP368 and their likely structures. All fragments above the blue dotted line are further highlighted in orange and will be observed in more detail

The extracted ion chromatogram (XIC) and MS² spectrum for the AMP-derived penicilloic acid in comparison to Arsand et al. 2018 is nearly the same (Arsand et al.. 2018). The typical fragmentation products of TP368 are 324 (C15H22N3O3S+), 307 (C15H19N2O3S+) and 279 (Arsand et al.. 2018). The fragments generated can also be created through the fragmentation of TP 324, as Arsand et al. found out with transformation products of 307 and 279 (Arsand et al.. 2018). Figure S5 shows potential fragmentation pathways.


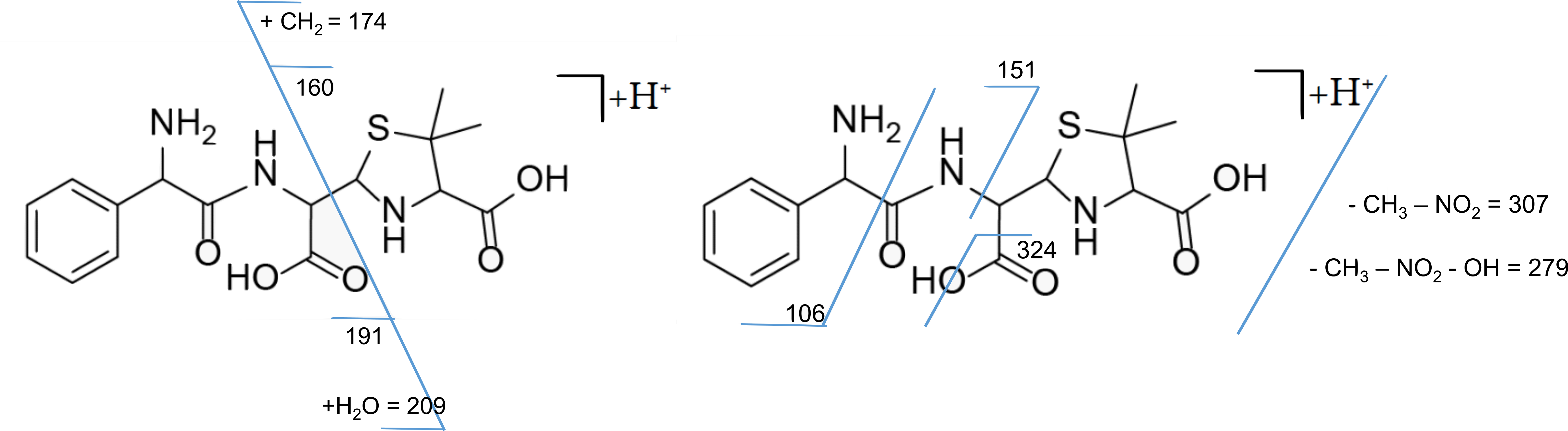


**Fig. S5** proposed generation of the different fragments of TP368 (inspired by Suwanrumpha & Freast 1989)

The TP324 arises through the decarboxylation (-CO2) of 368. The cutting off of the thiazolidine ring from 368 gives m/z = 160 and m/z = 191. The ion m/z = 191 is generated through the formation of a heterocyclic ring but if water is present the formation of m/z = 209 is also possible. The ion fragment m/z = 174 can result through the loss of water from fragment 192. Without this heterocyclic ring, m/z = 324 decomposes into m/z = 307 by removal of an amino group (-NH2). Furthermore, removal of a carbonyl group (-CO) results in the formation of the m/z = 279 fragment. The m/z = 307 fragment can also be formed directly from TP 368 by the loss of CH3 and NO2 and a fusion to a heterocyclic ring. The ion fragment m/z = 151 is generated through the splitting of the C-N linkage. Whereas m/z=106 consists out of the benzylamine group (Zhang et al.. 2014).

With the characterization of the TPs including further degradation fragments, effluents can be analyzed for the successful hydrolysis and thus inactivation of AMP.
